# Using structural MRI to identify bipolar disorders – 13 site machine learning study in 3020 individuals from the ENIGMA Bipolar Disorders Working Group

**DOI:** 10.1038/s41380-018-0228-9

**Published:** 2018-08-31

**Authors:** Abraham Nunes, Hugo G. Schnack, Christopher R. K. Ching, Ingrid Agartz, Theophilus N. Akudjedu, Martin Alda, Dag Alnæs, Silvia Alonso-Lana, Jochen Bauer, Bernhard T. Baune, Erlend Bøen, Caterina del Mar Bonnin, Geraldo F. Busatto, Erick J. Canales-Rodríguez, Dara M. Cannon, Xavier Caseras, Tiffany M. Chaim-Avancini, Udo Dannlowski, Ana M. Díaz-Zuluaga, Bruno Dietsche, Nhat Trung Doan, Edouard Duchesnay, Torbjørn Elvsåshagen, Daniel Emden, Lisa T. Eyler, Mar Fatjó-Vilas, Pauline Favre, Sonya F. Foley, Janice M. Fullerton, David C. Glahn, Jose M. Goikolea, Dominik Grotegerd, Tim Hahn, Chantal Henry, Derrek P. Hibar, Josselin Houenou, Fleur M. Howells, Neda Jahanshad, Tobias Kaufmann, Joanne Kenney, Tilo T. J. Kircher, Axel Krug, Trine V. Lagerberg, Rhoshel K. Lenroot, Carlos López-Jaramillo, Rodrigo Machado-Vieira, Ulrik F. Malt, Colm McDonald, Philip B. Mitchell, Benson Mwangi, Leila Nabulsi, Nils Opel, Bronwyn J. Overs, Julian A. Pineda-Zapata, Edith Pomarol-Clotet, Ronny Redlich, Gloria Roberts, Pedro G. Rosa, Raymond Salvador, Theodore D. Satterthwaite, Jair C. Soares, Dan J. Stein, Henk S. Temmingh, Thomas Trappenberg, Anne Uhlmann, Neeltje E. M. van Haren, Eduard Vieta, Lars T. Westlye, Daniel H. Wolf, Dilara Yüksel, Marcus V. Zanetti, Ole A. Andreassen, Paul M. Thompson, Tomas Hajek, for the ENIGMA Bipolar Disorders Working Group

**Affiliations:** 1 Department of Psychiatry, Dalhousie University, Halifax, Nova Scotia Canada; 2 Faculty of Computer Science, Dalhousie University, Halifax, Nova Scotia Canada; 3 Department of Psychiatry, Brain Center Rudolf Magnus, University Medical Center Utrecht, Utrecht University, Utrecht, The Netherlands; 4 Interdepartmental Neuroscience Program, University of California, Los Angeles, CA USA; 5 Imaging Genetics Center, Mark and Mary Stevens Neuroimaging and Informatics Institute, Keck School of Medicine of USC, University of Southern California, Marina del Rey, CA USA; 6 NORMENT KG Jebsen Centre, University of Oslo, Oslo, Norway; 7 Division of Mental Health and Addiction, Oslo University Hospital, Oslo, Norway; 8 Department of Psychiatric Research, Diakonhjemmet Hospital, Oslo, Norway; 9 Department of Clinical Neuroscience, Centre for Psychiatric Research, Karolinska Institutet, Stockholm, Sweden; 10 Centre for Neuroimaging and Cognitive Genomics (NICOG), Clinical Neuroimaging Laboratory, NCBES Galway Neuroscience Centre, College of Medicine Nursing and Health Sciences, National University of Ireland Galway, Galway, Ireland; 11 FIDMAG Germanes Hospitalàries Research Foundation, Barcelona, Spain; 12 Centro de Investigación Biomédica en Red de Salud Mental (CIBERSAM), Madrid, Spain; 13 Institute of Clinical Radiology, Medical Faculty – University of Muenster – and University Hospital Muenster, Muenster, Germany; 14 Department of Psychiatry, Melbourne Medical School, The University of Melbourne, Parkville, VIC Australia; 15 Hospital Clinic, University of Barcelona, IDIBAPS, CIBERSAM, Barcelona, Catalonia Spain; 16 Laboratory of Psychiatric Neuroimaging (LIM-21), Department and Institute of Psychiatry, Faculty of Medicine, University of São Paulo, São Paulo, Brazil; 17 Center for Interdisciplinary Research on Applied Neurosciences (NAPNA), University of São Paulo, São Paulo, Brazil; 18 MRC Centre for Neuropsychiatric Genetics and Genomics, Cardiff University, Cardiff, UK; 19 Department of Psychiatry, University of Münster, Münster, Germany; 20 Research Group in Psychiatry, Department of Psychiatry, Faculty of Medicine, Universidad de Antioquia, Medellín, Antioquia Colombia; 21 Department of Psychiatry and Psychotherapy, Philipps-University Marburg, Marburg, Germany; 22 NeuroSpin, CEA, Paris-Saclay, Gif sur Yvette, France; 23 Department of Neurology, Oslo Universisty Hospital, Oslo, Norway; 24 Department of Psychiatry, University of California, San Diego, La Jolla, CA USA; 25 Desert-Pacific Mental Illness Research, Education, and Clinical Center, VA San Diego Healthcare System, La Jolla, CA USA; 26 Departament de Biologia Evolutiva, Ecologia i Ciències Ambientals, Facultat de Biologia, Universitat de Barcelona, Barcelona, Spain; 27 Cardiff University Brain Research Imaging Centre, Cardiff University, Cardiff, UK; 28 Neuroscience Research Australia, Sydney, NSW Australia; 29 School of Medical Sciences, University of New South Wales, Sydney, NSW Australia; 30 Department of Psychiatry, Yale University, New Haven, CT USA; 31 Olin Neuropsychiatric Research Center, Institute of Living, Hartford Hospital, Hartford, CT USA; 32 Institut Pasteur, Unité Perception et Mémoire, Paris, France; 33 INSERM U955 Team 15 ‘Translational Psychiatry’, University Paris East, APHP, CHU Mondor, Fondation FondaMental, Créteil, France; 34 Neuroscience Institute, University of Cape Town, Cape Town, South Africa; 35 Translational Neuroscience Group, Department of Psychiatry and Mental Health, Cape Town, South Africa; 36 School of Psychiatry, University of New South Wales, Sydney, NSW Australia; 37 Department of Psychiatry and Behavioural Sciences, University of New Mexico, Albuquerque, NM USA; 38 Mood Disorders Program, Hospital Universitario San Vicente Fundación, Medellín, Antioquia Colombia; 39 Department of Psychiatry, University of Texas Health Science Center at Houston, Houston, TX USA; 40 Psychosomatic Unit, Division of Mental Health and Dependence, Oslo University Hospital and University of Oslo, Oslo, Norway; 41 University of Oslo, Institute of Clinical Medicine, Oslo, Norway; 42 Black Dog Institute, Prince of Wales Hospital, Sydney, NSW Australia; 43 Research Group, Instituto de Alta Tecnología Médica (IATM), Medellín, Antioquia Colombia; 44 Department of Psychiatry, University of Pennsylvania, Philadelphia, PA USA; 45 Department of Psychiatry, SA MRC Unit on Risk & Resilience in Mental Disorders, University of Cape Town, Cape Town, South Africa; 46 Western Cape Department of Health, Valkenberg Hospital, Cape Town, Western Cape South Africa; 47 Department of Psychiatry, University of Vermont, Burlington, VT USA; 48 Department of Child and Adolescent Psychiatry/Psychology, Erasmus Medical Centre, Rotterdam, The Netherlands; 49 Department of Psychology, University of Oslo, Oslo, Norway; 50 Instituto de Ensino e Pesquisa, Hospital Sírio-Libanês, Sao Paulo, Brazil

**Keywords:** Diagnostic markers, Bipolar disorder

## Abstract

Bipolar disorders (BDs) are among the leading causes of morbidity and disability. Objective biological markers, such as those based on brain imaging, could aid in clinical management of BD. Machine learning (ML) brings neuroimaging analyses to individual subject level and may potentially allow for their diagnostic use. However, fair and optimal application of ML requires large, multi-site datasets. We applied ML (support vector machines) to MRI data (regional cortical thickness, surface area, subcortical volumes) from 853 BD and 2167 control participants from 13 cohorts in the ENIGMA consortium. We attempted to differentiate BD from control participants, investigated different data handling strategies and studied the neuroimaging/clinical features most important for classification. Individual site accuracies ranged from 45.23% to 81.07%. Aggregate subject-level analyses yielded the highest accuracy (65.23%, 95% CI = 63.47–67.00, ROC-AUC = 71.49%, 95% CI = 69.39–73.59), followed by leave-one-site-out cross-validation (accuracy = 58.67%, 95% CI = 56.70–60.63). Meta-analysis of individual site accuracies did not provide above chance results. There was substantial agreement between the regions that contributed to identification of BD participants in the best performing site and in the aggregate dataset (Cohen’s Kappa = 0.83, 95% CI = 0.829–0.831). Treatment with anticonvulsants and age were associated with greater odds of correct classification. Although short of the 80% clinically relevant accuracy threshold, the results are promising and provide a fair and realistic estimate of classification performance, which can be achieved in a large, ecologically valid, multi-site sample of BD participants based on regional neurostructural measures. Furthermore, the significant classification in different samples was based on plausible and similar neuroanatomical features. Future multi-site studies should move towards sharing of raw/voxelwise neuroimaging data.

## Introduction

Bipolar disorders (BDs) are lifelong conditions, which tend to start in adolescence or early adulthood and consequently rank among the leading causes of morbidity and disability worldwide [1, 2]. Despite substantial advances in our understanding of the neurobiology of BD, the diagnostic system in psychiatry continues to be based on description of behavioral symptoms. This often results in delayed or inaccurate diagnosis of BD [3–5], which in turn leads to delayed or ineffective treatment [6]. Objective, biological markers could aid significantly in the clinical management of mental disorders [7], might reduce stigma, facilitate research and expedite the development of new treatments [8].

Brain imaging offers the unique ability to non-invasively investigate brain structure and function. Previous brain-imaging meta-analyses and large-scale multi-site studies have demonstrated that adults with BD had robust and replicable neurostructural alterations in subcortical, that is, hippocampus, amygdala, thalamus [9–11], as well as cortical regions, including inferior frontal gyrus, precentral gyrus, fusiform gyrus, middle frontal cortex [12–14]. Despite these advances and the relatively broad availability, the diagnostic potential of magnetic resonance imaging (MRI) in psychiatry has not been fully realized.

The translation of brain imaging from bench to the bedside has been hindered by the low sensitivity and specificity of between-group differences, by clinical heterogeneity and limited generalizability of findings from relatively small samples. The problem of low sensitivity and specificity may be overcome by novel analytical tools, such as machine learning (ML) [15, 16]. Traditional mass-univariate methods of MRI data analysis focus on localized and spatially segregated patterns of between-group differences [17]. The effect sizes of such changes (Cohen’s *d* = 0.15–0.29 [11, 14]) tend to be many times smaller than the effects needed for clinical application (Cohen’s *d* = 1.50–3.00 [18, 19]). In contrast, the ML techniques increase predictive power by targeting multivariate alterations distributed throughout the whole brain, which may better characterize the abnormalities found in psychiatric disorders [15, 20, 21]. Thus, ML brings neuroimaging analyses to the level of individual subjects, and with some caveats, potentially allows for diagnostic use. When previously applied to structural MRI, ML differentiated BD from control participants with accuracies between 59.5% [22] and 73.00% [23].

However, ML approaches typically require large samples to optimize the performance of the classifier, provide a generalizable snapshot of the studied disorder, decrease the risk of sampling effects and allow for application of rigorous cross-validation approaches [19]. Single-site studies may provide high site-specific accuracies [24], which, however, may not generalize across samples [25, 26]. Small studies may also yield a wide range of classification performances and inconsistencies in regions, which contribute to the overall classification [25–27]. Previous ML structural MRI studies in BD have typically included <50 BD participants recruited in a single site [23, 28–34]. The largest currently available neurostructural ML studies investigated 128–190 BD and 127–284 control participants [35–37], from up to two sites [22, 23, 38].

Large, multi-site datasets will necessarily be more heterogeneous than single site, carefully controlled samples. In fact, heterogeneity is one of the defining characteristics of big-data [39]. Single-site studies with rigorous inclusion/exclusion criteria may help isolate sources of heterogeneity, but they will represent only a small fraction of the “patient space.” In contrast, a large, multi-site study will primarily target generalizable alterations, which are shared among the participants, regardless of illness subtype, effects of treatment and other clinical variables. This is related to the fact that different sources of heterogeneity (i.e., presence of psychosis, neuroprogression, exposure to medications) affect different brain regions and often act in opposing directions [11–14, 40–42]. In addition, individual sources of heterogeneity, which are present only in some participants, are unlikely to systematically bias the findings in large, multi-site investigations. Thus, smaller, carefully controlled studies and large, multi-site datasets are complementary and ask different questions. BD is a broad and heterogeneous condition. Therefore, it is all the more important to quantify the extent to which ML can classify large, ecologically valid datasets based on neuroanatomy.

Researching generalizable brain alterations has only recently become possible through research consortia committed to aggregation and sharing of brain-imaging data across research groups. Despite the inherent limitations, retrospective data sharing initiatives create an optimal environment for application of ML strategies and for a fair and realistic estimation of the utility of MRI for classification of neuropsychiatric disorders. This approach has been utilized to improve predictive models of autism or Alzheimer dementia [26], but has not yet been applied to BD. The Enhancing Neuro Imaging Genetics through Meta-Analysis (ENIGMA) consortium is an international multi-cohort collaboration, which, by combining datasets from multiple sites, has allowed for more accurate testing of the reproducibility of disease effects in participants with schizophrenia [43], BD [11, 14] or major depression [44]. Due to the multi-site nature, methodological harmonization and access to some of the largest neuroimaging datasets to date, the ENIGMA platform provides an ideal opportunity to test ML on sufficiently large and generalizable samples.

In collaboration with the ENIGMA-BD Working Group, we applied ML to structural MRI data from 3020 participants recruited in 13 independent sites around the world. We attempted to differentiate BD from control participants based on brain structure. In addition, we studied the effects of different data handling strategies on classification performance, described the neuroanatomical features, which contributed to individual subject classification and investigated the effects of clinical variables on classification performance.

## Materials and methods

### Samples

The ENIGMA-BD Working Group brings together researchers with brain imaging and clinical data from BD participants and healthy controls [11, 14]. Thirteen of the sites from previously published ENIGMA studies [11, 14] provided individual subject data for this ML project. Each cohort’s demographics are detailed in Supplementary Table S1. Supplementary Table S2 lists the instruments used to obtain diagnosis and clinical information. Supplementary Table S3 lists exclusion criteria for study enrollment. Briefly, all studies used standard diagnostic instruments, including SCID (*N* = 10), MINI (*N* = 2) and DIGS (*N* = 1). Most studies (*N* = 7) included bipolar spectrum disorders, five studies included only BD-I and a single study only BD-II participants. Substance abuse was an exclusion criterion in 9/13 studies. Most studies (10/13) did not exclude comorbidities, other than substance abuse. A single study recruited medication naive participants. The remaining studies did not restrict medication use. Consequently, the sample is a broad, ecologically valid and generalizable representation of BD.

All participating sites obtained approval from their local institutional review boards and ethics committees, and all study participants provided written informed consent.

### Image processing and analyses

Structural T1-weighted MRI brain scans were acquired at each site and analyzed locally using harmonized analysis and quality control protocols from the ENIGMA consortium. Image acquisition parameters are listed in Supplementary Table S4. All groups used the same analytical protocol and performed the same visual and statistical quality assessment. These harmonized protocols were used in the previous publications by our group [11, 14] and they have been applied more broadly in large-scale ENIGMA studies of other disorders. Briefly, using a freely available and extensively validated FreeSurfer software, we performed segmentations and parcellations into 7 subcortical and 34 cortical gray matter regions per hemisphere (left and right), based on the Desikan–Killiany atlas. Visual quality controls were performed on a region of interest (ROI) level aided by a visual inspection guide including pass/fail segmentation examples. Diagnostic histogram plots were generated for each site and outlier subjects were flagged for further review. All ROIs failing quality inspection were withheld from subsequent analyses. Previous analyses from the ENIGMA-BD Working Group showed that scanner field strength, voxel volume and the version of FreeSurfer used for segmentation did not significantly influence the effect size estimates. Further details regarding these analyses, as well as forest plots of cortical and subcortical effect sizes from individual sites can be found here [11, 14].

### Data preprocessing

Input features were ROI cortical thicknesses (CT), surface area (SA) and subcortical volumes, a total of 150 features, and intracranial volume. As SA and CT are genetically distinct [45], influenced by different neurobiological mechanisms [46] and sometimes affected in opposite directions [47], we used both as input features. Prior to fitting of the ML classifier, we imputed missing data using mean values of the respective features, as well as centered and scaled each continuous feature.

Using statistical harmonization to reduce heterogeneity of data could improve accuracy [48], but at a cost to generalizability. Such approaches may compromise the train/test separation and may introduce additional assumptions, which are difficult to verify. Thus, in keeping with other studies [23, 38, 49], instead of statistical harmonization, we modeled between-site effects by using several different data handling strategies and investigated the association between relevant variables and classification accuracy, as described below.

### Support vector machine classifier

We a priori chose to use support vector machine (SVM [50]), which is the most frequently used ML method in psychiatric brain imaging [15, 51]. The present analyses implemented a linear kernel, because this limits the risk of overfitting, contains only a single parameter, see below, and the coefficients of a linear classifier can be interpreted as relative measures of feature importance. However, we also performed sensitivity analyses to determine the impact of using a non-linear kernel (radial basis function) on results. All ML analyses were implemented in the Python programming language v. 3.6 using the Scikit-Learn package v. 0.19 [52].

The linear kernel SVM has only a single parameter, C, which controls the trade-off between having zero training errors and allowing misclassifications. We decided to a priori fix the hyperparameter at C = 1, for the following reasons. First, this setting is a common choice in the existing literature [53–56]. Second, SVM performance is relatively robust to changes in C values [57]. Third, the decision to perform hyperparameter optimization has data costs, as one must perform a further nesting of cross-validation, resulting in smaller effective training sets [58]. Also, hyperparameter optimization involves many steps, which have not been standardized and which may contribute to vibration of effects, including introduction of further hyperparameters (of the optimizers), selection of the best objective function over which to optimize, selection of constraints over the hyperparameter being optimized and of the hyperparameter optimization algorithm. Nevertheless, we also performed sensitivity analyses to determine the impact of hyperparameter optimization in a nested cross-validation procedure, see Supplementary material.

As the features used in the present study are engineered (i.e., the feature set does not consist of raw, voxelwise images), we opted against further feature selection. This decision was also supported by the large sample size and the fact that we had 20 times more participants than features. Importantly, in the above-described methods, the SVM models are independent across folds and no statistical harmonization, model selection or comparison was done prior to splitting the samples into testing and training. Consequently, we have minimized the potential for information leak.

Classification performance was measured using standard metrics including accuracy, sensitivity, specificity, positive predictive value, negative predictive value and area under the receiver operating characteristic curve (ROC-AUC).

### Data handling

The first application of the above-described classifier was to the classification of cases versus controls in individual sites, referred to as site-level analyses. For each site, we fit an SVM and measured its performance using a stratified K-fold cross-validation procedure. This method is stratified insofar as the proportion of cases and controls (in respective folds) is similar in both training and validation sets. The number of folds was selected independently for each site, such that the validation set on each fold would have approximately 3 ( ±1) cases.

To further study how overall classification performance relates to different methods of data handling, we implemented three approaches. The first was a meta-analysis of diagnostic accuracy from site-level analyses, referred to as meta-analysis. This models the typical method of analyzing data in a multi-site collaboration [11, 14]. The meta-analyses were done using the hierarchical summary receiver operating characteristic, implemented in HSROC package v. 2.1.8 [59], in the R programming language, see Supplementary material.

Second, we evaluated the same linear SVM parameterization used in all other analyses on a leave-one-site-out (LOSO) cross-validation procedure, referred to as LOSO analyses. In each fold of cross-validation, one site’s data were completely excluded from the training partition. The SVM was then trained on the training partition and predictive performance was evaluated on the data from the held-out site.

Third, we fit an SVM classifier to the data pooled across all sites, using the same linear SVM parameterization as in the site-level analyses, and the same cross-validation procedure. This yielded a total of 284-folds and is further referred to as aggregate subject-level analysis.

We corrected for the effects of imbalanced data in all analyses and thereby trained the SVM classifiers on an effectively balanced dataset. To do this, we implemented the Synthetic Minority Oversampling Technique with Tomek link [60, 61] using the imblearn package v. 0.3.0.dev0 [62], in the Python language v. 3.6. The computer code for the above-described analyses will be provided upon reasonable request.

### Feature importance

To determine feature importance, we plotted the SVM coefficients learned (over a total of K-folds per sample) based on the aggregated data and the SVM coefficients learned from the site with the highest ROC-AUC performance. To quantify whether similar features contributed to classification in different analyses, we computed Cohen’s kappa for agreement in ranking of feature coefficients of individual regions between these two models, see Supplementary material for details of this calculation.

### Investigation of clinical heterogeneity/potential confounding factors

We investigated whether any confounding factors contributed to the classification by examining the relationship between clinical/demographic variables and classification results using mixed-effects logistic regression – glmer function in the lme4 package of the R Statistical Programming Language [63]. Variables listed in Table 1 and intercepts were taken as random effects varying between sites about a group mean, see Supplementary material. For numerical stability, age, age of onset and duration of illness were scaled to have mean 0 and unit variance.

**Table 1:** Descriptive statistics of the whole sample

	Controls	Cases	p-Value
*N*	2167	853	
Age mean (SD)	34.89 (12.41)	37.43 (11.64)	< 0.001
Sex, *N* (%) female	1201 (55.4)	516 (60.5)	0.013
Diagnosis, *N* (%)			
BD-I	-	582 (68.63)	
BD-II	-	234 (27.59)	
BD-NOS	-	13 (1.53)	
SZA	-	19 (2.24)	
Treatment at the time of scanning, *N* (%)			
Li		265 (33.5)	
AED	-	339 (43.1)	
FGA	-	32 (4.1)	
SGA	-	313 (39.9)	
AD	-	281 (35.5)	
Mood state, *N* (%)			
Euthymic	-	475 (75.5)	
Depressed	-	131 (20.8)	
Manic	-	11 (1.7)	
Hypomanic	-	9 (1.4)	
Mixed	-	3 (0.5)	
Age of onset mean (SD)	-	22.36 (9.08)	
Duration of illness mean (SD)	-	14.64 (10.45)	
History of psychosis, *N* (%)	-	372 (61.1)	

*AD* antidepressants, *AED* antiepileptics, *BD-I* bipolar I disorder, *BD-II* bipolar II disorder, *BD-NOS* bipolar disorder not otherwise specified, *FGA* first-generation antipsychotics, *Li* lithium, *SD* standard deviation, *SGA* second-generation antipsychotics, *SZA* schizoaffective disorder

## Results

We included 3020 participants (853 BD cases and 2167 controls), see Table 1.

**Fig. 1:**
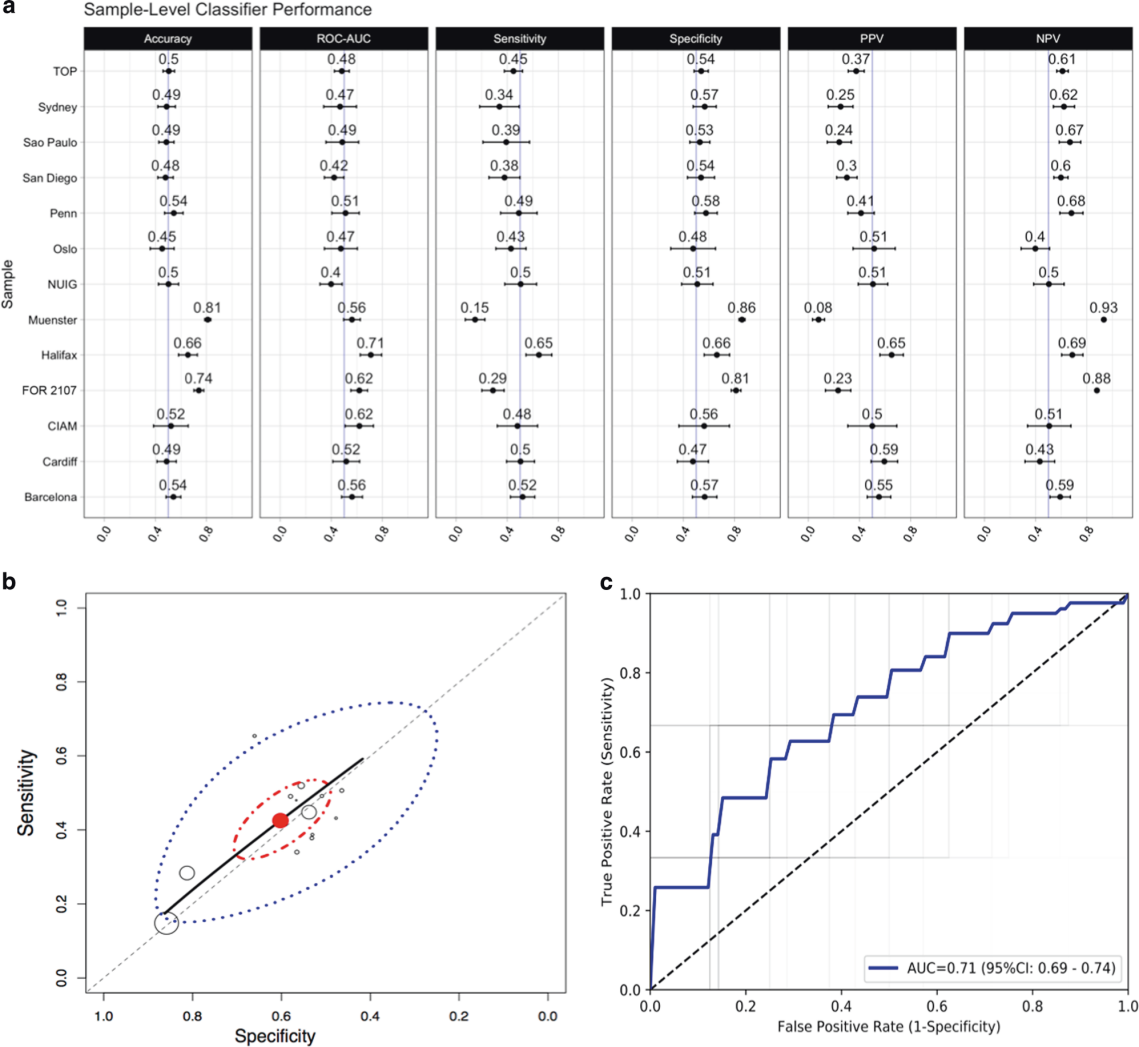
**a** Performance of SVM classifiers independently trained on each sample – mean with 95% confidence interval. Each row denotes a site in the data set, whereas each column denotes a specific performance metric. **b** Meta-analytic (summary) receiver operating characteristic (SROC) curves. Site-level sensitivity (Sn) and specificity (Sp) are empty circles of radius proportional to sample size. The red point is the median estimate of Sn and Sp. The solid black line is the SROC curve. Dashed diagonal represents chance performance. The red ellipse is the 95% posterior credible region, and the blue dashed line is the 95% posterior predictive region. **c** Receiver operating characteristic (ROC) curves for the aggregate subject-level analysis. Faint gray lines are the ROC curves for individual validation folds, and blue lines represent the mean ROC curve

The classification accuracy in individual sites ranged from 45.23% (95% confidence interval (95% CI) = 35.91–54.57) to 81.07% (95% CI = 78.68–83.46), see Fig. 1a. The classification performance was closely associated with the method of data handling. Meta-analysis of individual site results yielded the lowest performance, which did not exceed chance level, see Fig. 1b, Table 2. The LOSO cross-validation provided above chance classification, but performed worse than the aggregate subject-level analyses. Aggregating the data across sites yielded the highest and statistically significant classification performance, see Fig. 1c, Table 2.

**Table 2:** Summary of classification results from meta-analysis of sample-level classifiers, leave-one-site-out and aggregate subject-level analyses

Statistic	Meta-analysis	Leave-one-site-out	Aggregate subject-level
Accuracy (%)	-	58.67 (56.70–60.63)	65.23 (63.47–67.00)
ROC-AUC	-	60.92 (58.18–63.67)	71.49 (69.39–73.59)
Sensitivity (%)	42.60 (13.40–71.57)	51.99 (48.20–55.78)	66.02 (62.71–69.33)
Specificity (%)	59.14 (30.59–87.94)	64.85 (61.91–67.79)	64.90 (62.86–66.93)
PPV (%)	-	47.25 (37.67–56.84)	44.45 (42.04–46.86)
NPV (%)	-	67.67 (60.36–74.98)	83.73 (82.21–85.26)

Note that meta-analytic results of the HSROC package include only sensitivity and specificity of the overall meta-analytic classification. Results for meta-analytic summary are the posterior predictive value of the performance metric, reported as mean (95% credible interval; the Bayesian analog of 95% confidence intervals). Results for the aggregate subject-level and leave-one-site-out analyses are reported as mean and 95% confidence interval

*NPV* negative predictive value, *PPV* positive predictive value, *ROC-AUC* area under receiver operating characteristic curve

### Feature importance

**Fig. 2:**
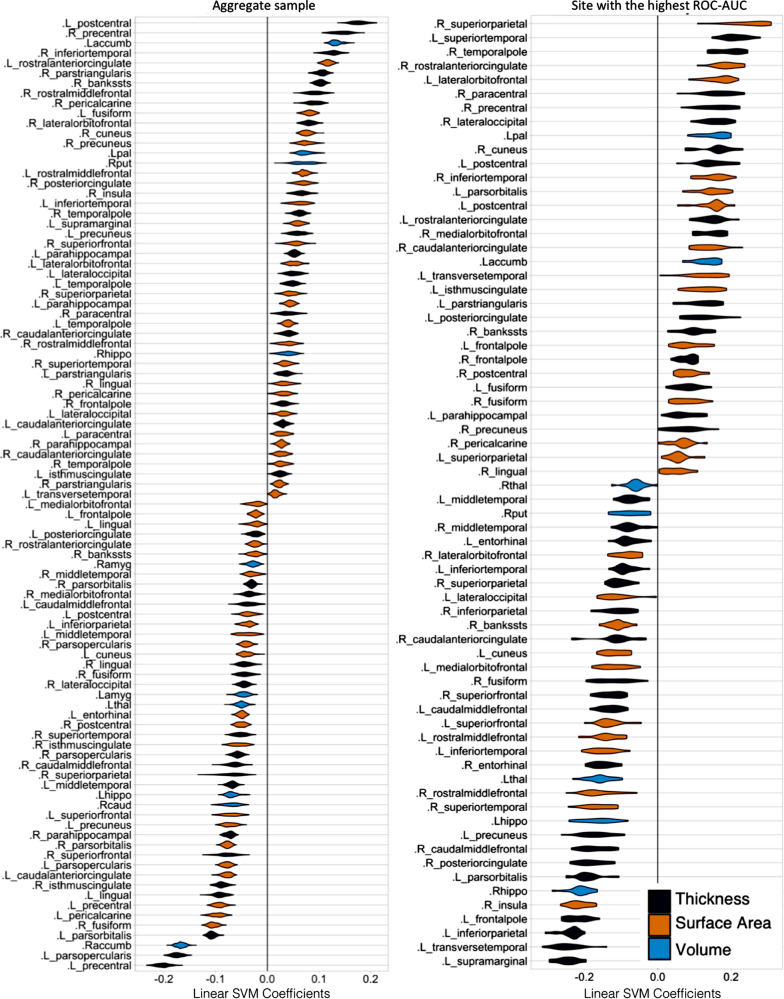
Violin plot of feature importance across cross-validation (CV) folds for aggregate subject-level analysis (left), and the site, which yielded the highest ROC-AUC (right). At each CV iteration, we extracted linear support vector machine (SVM) coefficients. The set of all coefficients from our SVM models are centered about 0. Deviation of coefficients from zero is an indication of the relative importance of individual features in the data. Features with positive and negative coefficients have positive and negative associations, respectively, with probability of classification as a case. The *y* axis lists variables for which SVM coefficients were strictly non-zero throughout all cross-validation iterations

Ranking of features, which contributed to classification in the site with the highest ROC-AUC and the aggregate subject-level analyses, see Fig. 2, showed substantial agreement (Cohen’s Kappa = 0.83, 95% CI = 0.829–0.831).

### Effects of clinical heterogeneity

Among BD participants in the aggregate subject-level analysis, both age (odds ratio (OR) = 1.4, 95% CI = 1.05–1.88, *p* = 0.02) and antiepileptic use (OR = 1.73, 95% CI = 1.07–2.78, *p* = 0.02) were positively and additively associated with correct classification. There was no association between correct classification and diagnostic subgroup, treatment with first-, second-generation antipsychotics, lithium (Li), age of onset, history of psychosis, mood state or sex, see Supplementary Table S5. Age was necessarily co-linear with duration of illness (*r*(782) = 0.66, *p* < 0.001), but there was no univariate association between the duration of illness and correct classification (OR = 1.18, 95% CI = 0.98–1.43, *p* = 0.09).

Treatment with anticonvulsants was negatively associated with Li treatment (OR = 0.39, 95% CI = 0.19–0.80, *p* = 0.01), but not with any other clinical features, see Supplementary Table S6.

In the whole sample, both age (OR = 1.46, 95% CI = 1.17–1.81, *p* < 0.001) and status (BD versus controls; OR = 1.60, 95% CI = 1.28–2.01, *p* < 0.001), but not sex (OR = 1.21, 95% CI = 0.99−1.48, p=0.06) were independently associated with being classified as a BD participant.

### Sensitivity analyses

Using the radial basis function kernel yielded accuracy of 68%, 95% CI = 67–69%. Hyperparameter optimization resulted in training set accuracy of 65.9%, 95% CI = 65.7–66.0 and testing set accuracy of 57.5%, 95% CI = 49.1–65.9. Thus, it is unlikely that substantial classification performance was sacrificed by forgoing kernel nonlinearity or hyperparameter optimization.

**Fig. 3:**
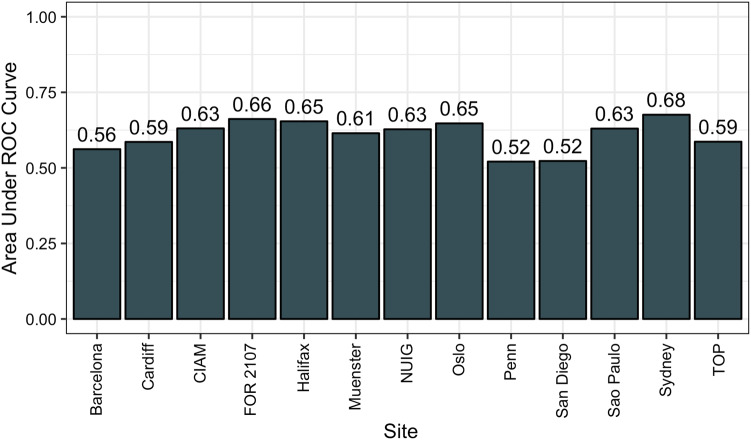
Bar plot of the area under the receiver operating characteristic curve (ROC-AUC) for the leave-one-site-out (LOSO) analyses. The sites listed along the *x* axis are those that were held-out at each fold

In the LOSO analysis, when we left out the sites with the highest ROC-AUC curves, i.e., Halifax, Marburg (FOR 2107), Cape Town (CIAM), we acquired ROC-AUC of 65.42%, 66.18%, 63.07%, respectively, see Fig. 3, which was comparable to the overall ROC-AUC of 60.92% in the LOSO. Thus, the overall results did not appear to be overly influenced by the best performing sites.

## Discussion

When applied to structural brain-imaging data, ML differentiated BD participants from controls with above chance accuracy even in a large and heterogeneous sample of 3020 participants from 13 sites worldwide. Aggregate analyses of individual subject data yielded better performance than LOSO or meta-analysis of site-level results. Despite the multi-site nature, ML identified a set of plausible brain-imaging features, which characterized individual BD participants and generalized across samples. Age and exposure to anticonvulsants were associated with greater odds of correct classification.

Previous studies employing raw structural MRI data have yielded accuracies between 59.50 and 73.00% [22, 23] for differentiating BD from control participants. A single study using results from automated segmentation reported accuracy below 60.00% [37]. Although direct comparison is complicated by methodological and sample size differences, the modest accuracies in previous studies are in keeping with our results. Thus, the presented findings appear realistic and there is little evidence for overfitting.

The classification performance in the aggregated dataset was significantly above chance level and the ROC-AUC of 71.49% (69.39–73.59) reached acceptable discrimination [64, 65]. However, the accuracy of 65.23% (95% CI = 63.47–67.00) fell short of the 80% threshold, which is deemed clinically relevant [66]. We need to consider several issues when interpreting these findings. BDs are difficult to diagnose even by standard methods. The Cohen’s kappa for reliability of the BD-I diagnosis is 0.56 and as low as 0.40 for BD-II [67]. In addition, the illness shows marked clinical and neurobiological heterogeneity [10, 12]. Perhaps most importantly, we worked with regional brain measures, not raw/voxelwise data. This approach necessarily involves some information loss in the feature engineering process. Analyses of experimenter-defined features are increasingly outperformed by models capable of learning abstractions from raw data alone [68]. Applying deep learning [69] to raw data would likely offer the greatest increase in classification accuracy.

This study provides important clues about the impact of data handling on the classification performance. As expected, the meta-analysis of individual site results, typically the first method of data analyses in multi-site collaborations, yielded the lowest accuracy, which did not exceed chance level. The LOSO analyses performed better than the meta-analytic approach, but worse than when individual subject data were aggregated and analyzed jointly. These differences in performance are likely related to the way each method handles the conditional relationships between the sites. Meta-analyses essentially model these relationships after the fact. The LOSO analyses are hindered by the fact that data are partitioned along some factor that is not random. In contrast, pooling of data allows for random partitioning and incorporates the relationships between the sites in their raw form. In addition, the classification performance is closely linked to the size of the training sample [49, 70], which increased from individual site through LOSO to aggregate analyses.

Thus, the empirical pattern of findings is convergent with theoretical prediction of how each of these methods should perform. It is also congruent with previous studies in autism [49], schizophrenia [70] and Alzheimer dementia [26], which also showed increasing performance with increasing size of the training set. It is a question whether this would also be the case in more heterogeneous conditions, such as major depression or anxiety disorders. Regardless, aggregate analyses provided the best classification performance in BD. Future multi-site brain-imaging studies should attempt to move towards sharing of individual subject data, not only site-level results.

The linear SVM kernel allowed us to visualize the contribution of individual regions to the overall classification. It is of note that the results of a backward model should not be used for localization [71]. We used this approach to broadly verify the neurobiological plausibility [26], not to infer pathophysiology. Our findings showed good validity, as many of the same regions, which have previously shown differences between groups of BD patients and controls, contributed to the classification on individual subject level, including hippocampus, amygdala [9–11], as well as cortical regions, such as inferior frontal gyrus [12, 14] and precentral gyrus [13].

In addition, we wanted to determine whether similar features were used for classification across different analyses. Indeed, there was a substantial agreement between the regions, which contributed to the classification in the site, which yielded the highest ROC-AUC and in the aggregate dataset, with Cohen’s Kappa of 0.83 (95% CI = 0.829–0.831). Furthermore, when we trained the classifier on data from all but the best performing sites, the classification performance did not drop below the overall accuracy in the LOSO analyses. Thus, individual sites did not markedly influence the overall findings. Taken together, these results suggest that the classification was based on a biologically plausible and generalizable neurostructural signature, which is shared among subjects in a large, multi-site sample. This is highly interesting, as existence of a generalizable biomarker is one of the key defining features of a diagnostic category [72].

We also investigated the effects of clinical/demographic variables on classification accuracy. Older age and anticonvulsant treatment were associated with greater odds of correct classification. The effect of age may be related to the fact that illness-related alterations may get worse with age/duration of illness [73]. Interestingly, similar association was noted in a meta-analysis of brain-imaging ML studies in schizophrenia [74]. These findings also broadly agree with another study, in which late-stage BD was easier to classify than early stage illness [36]. However, we did not find an association between accuracy of classification and duration of illness or age of onset.

The association with anticonvulsant treatment may reflect effects of illness or medications. Treatment with anticonvulsants was not associated with severity of illness, diagnostic category, mood state, age of onset or personal history of psychotic symptoms and thus did not appear to index a specific subgroup within BD. Interestingly, participants who were treated with anticonvulsants were less likely to also receive Li treatment. Perhaps, the neuroprotective effects of Li, which may normalize brain alterations in BD [10, 75] could presumably make the classification based on brain structure more difficult. However, Li treatment itself was not associated with classification accuracy. Previous studies have suggested that valproate, may negatively affect brain structure [76], which could contribute to correct differentiation of anticonvulsant treated from control participants. This was, however, not documented for lamotrigine, which is also frequently used in treatment of BD. Overall, the reasons why treatment with anticonvulsants and age were associated with easier classification are unclear and will be subject to future analyses.

A related question is whether the clinical/demographic heterogeneity confounded our findings and whether the age and/or treatment with anticonvulsants contributed more to the classification than the presence or absence of BD. Due to selection bias, heterogeneity is more likely to affect results in smaller studies [25]. The strength of a large, multi-center analysis is that it will primarily target the common alterations, which are generalizable to most participants and not individual sources of heterogeneity, which are present only in some [25]. In addition, both age and status were independently and additively associated with being classified as a BD participant in the whole sample. Also, within the site with the highest classification performance, BD participants and controls were balanced by age. In addition, 43.1% of patients in the whole sample were treated with anticonvulsants and yet, we reached a 66.02% sensitivity for correctly identifying BD participants. Last but not least, the sites with the highest proportion of anticonvulsant-treated participants (61.4%) and the highest discrepancy in age showed relatively low sensitivities of 49% and 29%, respectively. Thus, although certain clinical and demographic variables were associated with correct classification, it is unlikely that overall we were classifying participants based on the presence or absence of specific clinical/demographic variables, rather than the presence or absence of BD.

Our study has the following limitations. Due to differences in availability, we did not include other brain-imaging modalities or other types of data, that is, genetic, neurocognitive or biochemical. Access to raw data would allow us to use deep learning methods [68] or create a meta-model by combining classifiers trained on the local datasets [77]. However, currently there are significant practical and legal limitations to raw data sharing. The clinical heterogeneity and multi-site nature, which complicate traditional between-group comparisons,  allowed us to test the ML algorithms on a wide range of participants in a fair setting that better resembles a clinical situation. To achieve a clearer exposition and reduce methodological heterogeneity, we decided to use SVM. Previous studies have generally found minimal differences between “shallow” ML method [37]. As we worked with regional brain measures, not voxelwise data, we would not be able to fully exploit the power of deeper methods [78]. The depth and breadth of phenotyping are general issues in retrospective, multi-site data sharing collaborations. Specific sources of heterogeneity, that is, neuroprogression and comorbid conditions, may be particularly difficult to quantify. Addressing them would require a different research design. However, the large, multi-site sample, together with the exploratory analyses and examination of individual site results made it less likely that individual clinical characteristics systematically confounded the findings. Finally, attempting to differentiate BD from control participants is the first step before moving to more clinically relevant problems, such as differential diagnosis.

The key advantages of this study include the large, generalizable sample, access to individual subject data from 13 sites and the conservative and scalable nature of the analyses. This is currently the largest application of ML to brain-imaging data in BD, with up to two orders of magnitude, greater sample size than in previous studies. The unique nature of the dataset provides qualitative, not only quantitative advantages. Previous studies showed low stability of ML results with fewer than 130 participants [70], a threshold we exceeded 7–16 times. The multi-site dataset maximized the training set size, provided ecologically valid representation of the illness, allowed us to focus on common, BD-related alterations and for the first time apply the LOSO cross-validation in BD brain imaging. We completely separated the testing and training sets at each level of analysis, thus minimizing the risk of information leak, and specifically focused on maximizing generalizability/reducing the risk of overfitting. The study is an example of close international collaboration, which is one of the best ways, how to create optimal datasets for ML analyses.

## Conclusions

This study provides a realistic and fair estimate of classification performance, which can be achieved in a large, ecologically valid, multi-site sample of BD participants based on regional neurostructural measures. Although short of the 80% clinically relevant threshold the 65.23% accuracy, 71.49% ROC-AUC are promising, as we used an engineered feature set in a difficult to diagnose condition, which shows a marked clinical and neurobiological heterogeneity. In addition, similar, biologically plausible features contributed to classification in different analyses. Together these findings provide a proof of concept for a generalizable brain-imaging signature of BD, which can be detected on individual subject level, even in a large, multi-site sample. Although specific clinical/demographic characteristics, such age and anticonvulsant treatment, may affect classification, the clinical heterogeneity was not in the way of differentiating BD from control participants. Finally, we demonstrated that meta-analyses of individual site/study ML performances provide a poor proxy for results, which could be obtained by pooling of individual subject data. These findings are an important step towards translating brain imaging from bench to the bedside. They suggest that a multi-site ML classifier may correctly identify previously unseen data and aid in diagnosing individual BD participants. Application of deep learning to raw data might considerably increase the accuracy of classification.

## Electronic supplementary material

Supplemental Material
