# Supplementary material for: Using structural MRI to identify bipolar disorders – 13 site machine learning study in 3020 individuals from the ENIGMA Bipolar Disorders Working Group
Source: Mol Psychiatry. 2018 Aug 31;25(9):2130–43. doi: 10.1038/s41380-018-0228-9 (PMC7473838; doi:10.1038/s41380-018-0228-9)
Supplement: Supplementary file 1 — Supplemental Material [file 41380_2018_228_MOESM1_ESM.pdf]

SUPPLEMENTARY METHODS AND MATERIALS

Hyperparameter optimization

Although C=1 was our a priori choice, we also performed sensitivity analyses to determine the impact of hyperparameter optimization in a nested cross-validation procedure, see Supplementary material. The sensitivity analysis for effect of hyperparameter optimization was done under the following. The outer cross-validation loop was stratified-20-fold cross validation with roughly equal partitioning of cases and controls in each fold. On the training set (within each respective outer fold), we implemented another stratified-K-fold cross validation procedure (computed such that each validation fold had approximately 20 cases; total of 41 folds) and tuned the SVM hyperparameters with Bayesian Optimization, where the target metric for optimization was the F1-Score. The F1 score is a useful performance metric for imbalanced data problems (where the positive target class is relatively rare). The SVM hyperparameter that was optimized was C, and the feasible region was left broad on the closed interval [0.001, 1000]. We also specified that C=1 should be explored explicitly as a candidate solution (i.e. it was always considered by the hyperparameter optimizer).

Meta-analysis of diagnostic accuracy

We pooled the classification results from the site-level analyses, and performed joint meta-analysis of diagnostic accuracy using the HSROC package v. 2.1.8, (1) in the R programming language. Computation of a summary ROC curve was preferred to a simple averaging of the accuracy (or other performance measure) because it allows for a richer description of the uncertainty in the relationships between true and false positive rates that may be observed across sites. Hierarchical models for diagnostic meta analyses are recommended by the Cochrane collaboration (http://methods.cochrane.org/sdt/handbook-dta-reviews), and we chose a Bayesian implementation by virtue of the ability to estimate full posterior densities over estimates. The Gibbs sampling procedure in HSROC was implemented with 4 chains, with 50,000 iterations (discarding 10,000 for burn in, and using thinning of 2).

Cohen's Kappa for Rank Association

For each fold of cross validation with an SVM, we can extract a vector of coefficients that is of dimension $N_{features} \times 1$. We denote this vector as

$$C^k = \left(c^k_{x_1}, c^k_{x_2}, \dots, c^k_{x_{N_{features}}}\right)^{\top},$$

where $c^k_{x_i}$ is the SVM coefficient for the ith feature (x_i) during the kth fold of cross-validation. With this representation, we define the indicator function

$$\mathbb{I}\left[c_{x_i}^k < c_{x_j}^k\right] = \begin{cases} 1 & if\ c_{x_i}^k < c_{x_j}^k \\ 0 & otherwise \end{cases},$$

which returns a value of 1 if the coefficient of feature x_i was less than the coefficient of feature x_j at the kth fold of cross-validation. If we compute $\mathbb{I}$ for every pair of features, then we can preserve all ranking information within a binary matrix $R^k \in \{0, 1\}^{N_{features} \times N_{features}}$. The entry at the ith row and j^{th} column of R^k is

$$R_{ij}^k = \mathbb{I}\left[c_{x_i}^k < c_{x_j}^k\right].$$

Note that R^k does not explicitly record the distance in rankings between features (x_i, x_j). However, through enumeration of all pairwise binary rankings that information is preserved in R^k.

We now denote the feature ranking matrix for the kth fold of the aggregated analysis SVM as $R^{Agg,k}$, and the corresponding feature ranking matrix at the mth fold of the best site-level SVM as $R^{Site,k}$. The probability that these models agree in ranking of features x_i and x_j when comparing across all cross-validation folds is

$$P(Agreement_{km}) = P\left(R^{Agg,k}, R^{Site,m}\right) = \langle\ \mathbb{I}\left[R_{ij}^{Agg,k} = R_{ij}^{Site,m}\right]\rangle_{i\square}$$

and the chance level of agreement is

$$P(ChanceAgree_{km}) = P\left(R^{Agg,k}\right)P\left(R^{Site,m}\right) = \langle\ \mathbb{I}\left[R_{ij}^{Agg,k}\right]\rangle_{ij} \cdot \langle\ \mathbb{I}\left[R_{ij}^{Site,m}\right]\rangle_{ij}.$$

The $\langle\cdot\rangle_{ij}$ angles denote the expectation over all feature pairings (x_i, x_j). Thus, we can define a Cohen's Kappa measure for rank agreements as

$$\kappa_{km} = \frac{P(Agreement_{km}) - P(ChanceAgree_{km})}{1 - P(ChanceAgree_{km})},$$

from which a mean and variance can be computed respectively as

$$\bar{\kappa} = \frac{1}{KM}\sum_{k=1}^{K}\sum_{m=1}^{M}\kappa_{km}\ ,\quad \sigma_\kappa^2 = \frac{1}{KM}\sum_{k=1}^{K}\sum_{m=1}^{M}(\kappa_{km} - \bar{\kappa})^2\ .$$

It follows that the 95% confidence interval about the mean is

$$\bar{\kappa} \pm 1.96\,\frac{\sigma_\kappa}{\sqrt{KM}}.$$

Investigation of clinical heterogeneity/potential confounding factors

We investigated whether any confounding factors contributed to the model, by examining the relationship between clinical variables and the probability of correct classification using mixed-effects logistic regression (glmer function in the lme4 package of the R Statistical Programming Language (2). Variables listed in table 1 and intercepts were taken as random effects varying between sites about a group mean, see Supplementary material

For numerical stability, age and age of onset were scaled to have mean 0 and unit variance. Fixed-effects correlations and random-effects distributions were inspected graphically to ascertain satisfaction of model assumptions. Parameter estimation was done using bounded optimization by quadratic optimization, and resulting estimates reported as odds ratios. Approximation of the model's degrees of freedom and computation of p-values for parameter estimates was done using Satterthwaite's approximation in the lmerTest package in *R* (3;4). The significance threshold was set a priori to alpha=0.05.

SUPPLEMENTARY TABLES

Supplementary Table S1: Descriptive statistics of included samples. Abbreviations: AD antidepressants, AED antiepileptics, BD bipolar disorders, SZA schizoaffective disorder, FGA first generation antipsychotics, SGA second generation antipsychotics, Mood state legend: 1 = Euthymic, 2 = Depressed, 3 = Manic, 4 = Hypomanic, 5 = Mixed
p value legend * p<0.05, ** p<0.01, *** p<0.001

	Barcelona		Cardiff		CIAM		FOR 2107		Halifax		Muenster		NUIG	
	Control	Case	Control	Case	Control	Case	Control	Case	Control	Case	Control	Case	Control	Case
**N**	117	102	56	79	30	25	432	67	56	52	749	54	55	59
**Age**	41.3 (9.6)	41.7 (9.4)	38.1 (9.5)	40.1 (8.6)	26.6 (5.0)	29.6 (5.2)*	31.4 (12.1)	[illegible]2.9 (11.6)**[illegible]	41.5 (12.3)	46.2 (13.8)	35.2 (12.1)	38.2 (11.8)	41.7 (9.8)	41.7 (9.7)
**Female**	62 (53.0)	57 (55.9)	37 (66.1)	53 (67.1)	14 (46.7)	10 (40.0)	269 (62.3)	43 (64.2)	37 (66.1)	34 (65.4)	424 (56.6)	28 (51.9)	27 (49.1)	28 (47.5)
**Full Dx**														
**BD-I**		102 (100.0)		34 (43.0)		25 (100.0)		28 (41.8)		32 (61.5)		54 (100.0)		59 (100.0)
**BD-II**		0 (0.0)		45 (57.0)		0 (0.0)		20 (29.9)		19 (36.5)		0 (0.0)		0 (0.0)
**BD-NOS**		0 (0.0)		0 (0.0)		0 (0.0)		0 (0.0)		1 (1.9)		0 (0.0)		0 (0.0)
**SZA**		0 (0.0)		0 (0.0)		0 (0.0)		19 (28.4)		0 (0.0)		0 (0.0)		0 (0.0)
**Li**		67 (70.5)		19 (24.1)		11 (44.0)		11 (16.7)		26 (50.0)		15 (28.3)		44 (75.9)
**AED**		49 (51.6)		34 (43.0)		15 (60.0)		17 (25.8)		22 (42.3)		20 (37.7)		26 (44.1)
**FGA**		6 (6.3)		1 (1.3)		8 (32.0)		2 (3.0)		0 (0.0)		2 (3.8)		4 (6.9)
**SGA**		51 (53.7)		29 (37.2)		10 (40.0)		38 (57.6)		17 (32.7)		31 (58.5)		4 (6.8)
**AD**		22 (23.4)		41 (51.9)		2 (8.0)		26 (39.4)		19 (36.5)		33 (62.3)		13 (22.0)
**Mood State**														
**1**		100 (98.0)		78 (98.7)		25 (100.0)		12 (21.8)		52 (100.0)		0 (0.0)		59 (100.0)
**2**		2 (2.0)		0 (0.0)		0 (0.0)		27 (49.1)		0 (0.0)		54 (100.0)		0 (0.0)
**3**		0 (0.0)		0 (0.0)		0 (0.0)		9 (16.4)		0 (0.0)		0 (0.0)		0 (0.0)
**4**		0 (0.0)		1 (1.3)		0 (0.0)		6 (10.9)		0 (0.0)		0 (0.0)		0 (0.0)
**5**		0 (0.0)		0 (0.0)		0 (0.0)		1 (1.8)		0 (0.0)		0 (0.0)		0 (0.0)
**Onset**		25.8 (8.6)		19.0 (7.0)		21.5 (4.4)		24.9 (11.6)		21.8 (7.15)		26.2 (8.8)		28.5 (8.2)
**Hx Psychosis**		74 (76.3)		13 (21.3)		25 (100.0)		-		20 (38.5)		-		56 (94.9)

	Oslo		Penn		San Diego		Sao Paulo		Sydney		TOP		P-Values (Inter-Site)
	Control	Case	Control	Case	Control	Case	Control	Case	Control	Case	Control	Case	
**N**	44	44	88	53	79	45	66	31	92	50	303	192	
**Age**	31.2 (9.1)	34.4 (7.3)	37.4 (14.3)	[illegible]9.7 (10.1)**[illegible]	53.2 (13.8)	[illegible]5.2 (10.2)**[illegible]	26.9 (5.5)	29.3 (7.1)	22.6 (3.7)	25.0 (3.7)***	34.8 (9.7)	34.9 (11.6)	***
**Female**	26 (59.1)	32 (72.7)	44 (50.0)	32 (60.4)	44 (55.7)	33 (73.3)	22 (33.3)	20 (64.5) **	51 (55.4)	34 (68.0)	144 (47.5)	112 (58.3)*	
**Full Dx**													***
**BD-I**		0 (0.0)		42 (79.2)		45 (100)		16 (51.6)		28 (56.0)		117 (62.6)	
**BD-II**		44 (100.0)		7 (13.2)		0		15 (48.4)		22 (44.0)		62 (33.2)	
**BD-NOS**		0 (0.0)		4 (7.5)		0		0 (0.0)		0 (0.0)		8 (4.3)	
**SZA**		0 (0.0)		0 (0.0)		0		0 (0.0)		0 (0.0)		0 (0.0)	
**Li**		3 (6.8)		19 (37.3)		-		0 (0.0)		15 (30.0)		35 (18.7)	***
**AED**		20 (45.5)		26 (51.0)		-		0 (0.0)		27 (61.4)		83 (44.4)	***
**FGA**		2 (4.5)		0 (0.0)		-		0 (0.0)		0 (0.0)		7 (3.7)	***
**SGA**		4 (9.1)		23 (45.1)		-		0 (0.0)		13 (29.5)		93 (49.7)	***
**AD**		17 (38.6)		14 (27.5)		-		0 (0.0)		22 (44.0)		72 (38.5)	***
**Mood State**													***
**1**		24 (54.5)		6 (15.0)		-		-		50 (100.0)		69 (100.0)	
**2**		19 (43.2)		29 (72.5)		-		-		0 (0.0)		0 (0.0)	
**3**		0 (0.0)		2 (5.0)		-		-		0 (0.0)		0 (0.0)	
**4**		1 (2.3)		1 (2.5)		-		-		0 (0.0)		0 (0.0)	
**5**		0 (0.0)		2 (5.0)		-		-		0 (0.0)		0 (0.0)	
**Onset**		16.0 (5.8)		19.3 (8.7)		-		24.8 (7.9)		15.1 (3.6)		22.0 (9.3)	***
**Hx Psychosis**		-		34 (70.8)		-		25 (80.6)		18 (36.7)		107 (57.2)	***

Supplementary Table S2. Diagnosis and medication information.

Sample	Instrument for diagnosing bipolar disorders	Method for obtaining medication information
Barcelona	Structured Clinical Interview for DSM-IV and Research Diagnostic Criteria (RDC).	Detailed clinical interview and review of case notes.
Cardiff	Consensus Consultant Diagnosis and Mini International Neuropsychiatric Interview (MINI)	Patient interview
CIAM (South Africa)	Structured Clinical Interview for DSM-IV for Axis I Diagnoses	Patient interview and Hospital records
Halifax	Structured Clinical Interview for DSM-IV for Axis I Diagnoses; (Halifax): Participants were recruited from patients followed up at a specialized Mood Disorders Program at Dalhousie University, Halifax, NS. The Program is a tertiary care clinic providing consultation services to family physicians and community psychiatrists and following up patients with BD. The diagnostic interviews were performed by pairs of clinicians, according to the Schedule for Affective Disorders and Schizophrenia, Lifetime version (SADS-L) and diagnoses were made according to DSM-IV criteria.	Questionnaire with self and interviewer reporting, in part using validated instruments; (Halifax): Patients had regular follow ups at the clinic, including monitoring of Li levels at least twice per year. Furthermore, we established illness course and treatment response to Li using NIMH life charts (NIMH-LCMTM)
Malt-Oslo	Mini-International Neuropsychiatric Interview (MINI), DSM-IV criteria version 5.0.	Stanley Foundation Network Entry Questionnaire (NEQ).
FOR2107	Structured Clinical Interview for DSM-IV for Axis I Diagnoses	self-report and hospital records
Muenster Neuroimaging Cohort	Structured Clinical Interview for DSM-IV for Axis I Diagnoses	self-report and hospital records
NUIG	Structured Clinical Interview for DSMIV-TR-Patient Edition for patients and SCID_NP for controls	Detailed clinical interview outlining dose and duration of all psychotropic medication, supplemented by clinical notes where necessary.
Sao Paolo	Structured Clinical Interview for DSM-IV for Axis I Diagnoses	Self-report and clinical records
Sydney	Diagnostic Interview for Genetic Studies (for 22-30 year-olds); Kiddie-SADS (for 12-21 year-olds)	Patient interview and Adult Health Screening questionnaire
TOP	Structured Clinical Interview for DSM-IV for Axis I Diagnoses	Structured patient interview, and hospital records
UCSD	Expanded Structured Clinical Interview for DSM-IV for Axis I Diagnoses	Self-report and clinical records

**UPENN**	**Structured Clinical Interview for DSM-IV for Axis I Diagnoses**	**Combination of self-report and clinician report**

Supplementary Table S3. Inclusion and exclusion criteria used by each site.

Sample	Criteria for Inclusion/Exclusion
**Barcelona**	All patients with bipolar disorder were right handed. Exclusion criteria were age younger than 18 or older than 65 years, history of neurological disease or brain trauma, and alcohol/substance abuse in the 12 months prior to participation. Patients were also required to have a current IQ in the normal range (>70). All patients were diagnosed using DSM-IV and Research Diagnostic Criteria (RDC), based on a detailed clinical interview and review of case notes.<br>All healthy controls met the same exclusion criteria as the patients, and they were interviewed and excluded if they reported a history of mental illness and/or treatment with psychotropic medication other than non-regular use of benzodiazepines or similar drugs for insomnia. They were also questioned about family history of mental illness and excluded if a first-degree relative had experienced symptoms consistent with major psychiatric disorder and/or had received any form of in- or outpatient psychiatric care.<br>The healthy controls were selected to be matched with the patients on demographic variables and on premorbid IQ.
**Cardiff**	Inclusion criteria: age >18, <60; positive diagnosis of BD-I or BD-II; Euthymia (absence of any significant mood episode for 2 months prior scanning, unchanged drug treatment for the same period, and HAM-D and YMRS <10 on day of scan); no personal history of psychotic disorders or Borderline Personality Disorder; < 1 year history of OH or substance abuse/dependence; no contraindications for MRI scan.
**CIAM (South Africa)**	Bipolar disorder participants were required to meet a diagnosis of bipolar I disorder with a significant history of psychosis. Between the ages of 19 and 40. Stable outpatients were recruited, and did not meet either mood polarity at the time of scanning. Were compatible for MRI and EEG imaging. Exclusion included history of epilepsy or seizures, which was an exclusion for EEG testing performed. Exclusion of any participants if presented with a significant/chronic general medication condition, e.g.. HIV, diabetes I/II, highblood pressure. Further for female participants no current/recent/suspected pregnancy or current lactation were allowed to participate.

**Halifax**	Inclusion criteria. The BD patients (both Li and non-Li groups) had to have: (i) a diagnosis of bipolar I or II disorder made by a psychiatrist using the SCID; (ii) at least 10 years of illness; (iii) a history of at least five episodes of illness (including manic, depressive, or mixed episodes); (iv) current Hamilton Depression Rating Scale, 17-item version (HAM-D-17) score < 7; (v) current Young Mania Rating Scale (YMRS) score < 5; (vi) current Clinical Global Impressions Scale–Bipolar (CGI-BP) score < 3; and (vii) a period of euthymia for at least four months prior to scanning, as aside from state-related factors, patients in acute episodes may present with additional difficult to control confounding variables, including recent medication change or substance abuse. The non-Li group had to have less than three months of lifetime Li exposure, more than 24 months prior to the scanning. The Li group had to have a current Li treatment lasting a minimum of 24 months. Exclusion criteria. Individuals from any of the three groups were excluded if they met any of the magnetic resonance imaging (MRI) exclusion criteria or had any serious medical illness (e.g., brain injury, Cushings disease, or conditions treated with corticosteroids). Individuals with BD were excluded if they had: (i) more than one lifetime course of electroconvulsive therapy (ECT) or ECT in the previous 12 months; (ii) comorbid psychiatric disorders, and / or personality disorder; (iii) active substance abuse in the previous 12 months; (iv) significant change in their medication in the previous three months; or (v) current psychotic features or acute suicidality. Individuals from the non-Li group were excluded if they had: (i) Li exposure < 2 years before the scanning; or (ii) lifetime Li exposure of more than three months. The neuropsychiatrically healthy individuals were excluded if they had a personal history of psychiatric disorders. (Halifax) Diabetes Study: The subjects with BD were required to 1) have the diagnosis of bipolar I or II disorder made by a psychiatrist; and 2) be at least 18 years of age. Patients were excluded if they had 1) the diagnosis of organic mood disorder; 2) mood disorder not otherwise specified; or 3) more than one lifetime course of electroconvulsive therapy or electroconvulsive therapy within the last 6 months. The neuropsychiatrically healthy, euglycemic subjects were excluded if they had 1) a personal history of psychiatric disorders; or 2) T2DM. Subjects from any group were excluded if they 1) met any magnetic resonance imaging (MRI) exclusion criteria; 2) suffered from substance abuse in the last 12 months; had a history of 3) neurodegenerative disorders; or 4) cerebrovascular disease/stroke, as we were interested in the more subtle T2DM-related neuronal changes. Halifax High Risk Study: Families were identified through adult probands with BD, who had participated in 1) previous genetic and high-risk studies for the Halifax sample. Only the offspring from these families, not the probands, were a part of the MRI study. The offspring from BD parents were divided into two subgroups: 1) the Unaffected HR group, which consisted of 50 offspring with no lifetime history of psychiatric disorders. These individuals were at an increased risk for BD because they had one parent affected with a primary mood disorder. 2) The Affected Familial group, which consisted of 36 offspring who met criteria for a lifetime Axis I diagnosis of mood disorders (i.e., a personal history of at least one episode of depression, hypomania, or mania meeting full DSM-IV criteria). When available, we recruited more than one offspring per family. From this study, we provided data only from patients who had a personal history of bipolar disorder.
**Malt-Oslo**	Inclusion criteria patients: A DSM-IV diagnosis of bipolar disorder type II. Exclusion criteria healthy controls: Controls with previous or current psychiatric illness were excluded from the study. The exclusion criteria for all participants were: A.) age younger than 18 or older than 50 years; B.) previous head injury with loss of consciousness for more than 1 minute; C.) history of neurological or other severe chronic somatic disorder; D.) pregnancy; E.) metallic implants.
**FOR2107**	Inclusion criteria: age 18-65 years; patients were diagnosed of bipolar I disorder by SCID-Interview, currently depressed, (hypo)manic or remitted.<br>Exclusion criteria all: any MRI contraindications; any neurological abnormalities.<br>Exclusion criteria controls: any current or former psychiatric disorder;<br>Exclusion criteria patients: substance dependence or current benzodiazepine treatment (wash out of at least three half-lives before study participation)
**Muenster Neuroimaging Cohort**	Inclusion criteria: age 17-65 years; patients were diagnosed of bipolar I disorder by SCID-Interview, currently depressed (HAMD >= 18);<br>Exclusion criteria all: any MRI contraindications; any neurological abnormalities;<br>Exclusion criteria controls: any current or former psychiatric disorder;<br>Exclusion criteria patients: substance-related disorders or current benzodiazepine treatment (wash out of at least three half-lives before study participation), and former electroconvulsive therapy

**NUIG**	Inclusion criteria: DSM-IV diagnosis of bipolar disorder (patients); age >18 and <60. Exclusion criteria: history of neurological illness (comorbid); lifetime DSM-IV axis 1 disorder or family history of psychotic or affective disorder in first- or second-degree relatives (controls); history of substance and/or alcohol misuse in the past year; learning disability; recent oral steroid use.
**Sao Paolo**	Treatment-naive individuals fulfilling DSM-IV criteria for BD type I or II, at any phase of the illness. Heathly controls were free of any mental disorder and had no history of mood or psychotic disorders among first-degree relatives. Other inclusion/ exclusion criteria for both study groups: Aged between 18 - 45 years; Free of substance use disorders (lifetime); Right-handed; Absence of neurological disorders or any organic disorders that could affect the central nervous system, No history of head trauma with loss of consciousness; No contraindication for MRI scanning.
**Sydney**	Bipolar disorder participants meet DSM-IV criteria for either bipolar I or bipolar II disorder. Control participants meet criteria if no parent or sibling had bipolar I or II disorder, recurrent major depression, schizoaffective disorder, schizophrenia, recurrent substance abuse or any past psychiatric hospitalisation; and no parent with a first degree relative had a past mood disorder hospitalisation or history of psychosis. All subjects in are aged between 12 and 30 years. For those aged between 12 and 21 an adapted version of the Schedule for Affective Disorders and Schizophrenia for School-Age Children – Present and Lifetime Version (K-SADS-BP) was developed specifically for use in the US-Australia collaborative study of young people at genetic risk for BD. For participants aged between 22 and 30 the DIGS (Version 4) is used to measure the current and lifetime presence of axis I DSM-IV disorders.
**TOP**	Inclusion criteria: Patients with bipolar spectrum disorder between ages 18-65 recruited from psychiatric departments and outpatient clinics in Oslo as part of the Thematically Organized Psychosis (TOP) Research study. Exclusion criteria were: IQ < 70, a condition better accounted for by substance abuse or somatic illness, having a brain illness or a previous moderate/severe head injury. General: All participants signed a written informed consent. The study was approved by the Regional Committee for Medical Research Ethics and the Norwegian Data Inspectorate, and conducted in accordance with the Helsinki declaration.
**UCSD**	Inclusion criteria for patients: DSM-IV diagnosis of BD and onset of first mood episode between age 13 to 35 years. Exclusion criteria for patients: Current depressive or manic episode as determined by DSM-IV criteria or significant residual mood or psychotic symptoms, change of medication or dose in past 6 weeks, other co-morbid Axis I disorder (anxiety disorder allowed if no symptoms or treatment within 1 year), current or recent (past 6 months for abuse, past 12 months for dependence) diagnosis of substance abuse or dependence, history of head injury with loss of consciousness for > 30 minutes, left handedness, history of neurological disorder (e.g., seizure disorder, Parkinson's or Alzheimer's disease, stroke), history of diabetes, uncontrolled hypertension, contraindications for MRI scanning (e.g., metal in the body, weight over 300 lbs, claustrophobia, difficulty lying still, pregnancy), native language other than English, conservatorized. Exclusion criteria for healthy individuals: current Axis I disorder as determined by the Mini-International Neuropsychiatric Interview (MINI, first-degree relatives with bipolar disorder, unipolar depression, or schizophrenia, and all the non-psychiatric exclusion criteria listed above.)
**UPENN**	Inclusion criteria: DSM-IV diagnosis of bipolar disorder; clinically stable, without recent (< 2 week) clinically significant changes in medication type or dose. As long as they are clinically stable, patients may have active illness symptoms, including hallucinations, mood elevation, irritability, or depression. Patients deemed to be at elevated risk of self-harm or violence will be excluded from the study. Age >18 and <60, proficiency in English. Exclusion criteria: significant medical or neurological illness that may effect brain function or impede participation; substance abuse within 6 months of participation; developmental disorders or mental retardation; pregnancy; history of pathological gambling; no contraindications for MRI.

Supplementary Table S4. Image acquisition and processing details by site.

Site	Sequence	Field Strength	Acquisition Direction	# of Slices	Slice Gap	Voxel Size (mm3)	TI	TE	TR	Flip Angle	Citation
**Barcelona**	3D T1-weighted enhanced fast gradient echo (EFGRE 3D)	1.5T GE Signa	Axial	180	0mm	0.47×0.47×1	710ms	3.93ms	2000 ms	15	(5;6)
**Cardiff**	3D T1-weighted fast spoiled gradient recall (3D FSPGR)	3T GE HDx	Axial	172	0mm	1x1x1	450ms	3ms	7.9ms	20	(7)
**CIAM (South Africa)**	3D T1-weighted magnetization prepared rapid acquisition gradietn echo (MPRAGE)	3T Siemens Allegra	Sagittal	128	0mm	1.3×1.0×1.3	1100 ms	1.53ms; 3.21ms; 4.89ms; 6.57ms	2530 mm	7	(8)
**Halifax**	3D T1-weighted spoiled gradient recalled acquisition in steady state	1.5T GE Signa	Coronal	124	1.5mm	0.9375x0.9375x1.5	0ms	5ms	25ms	40	(9-11)
**Malt-Oslo**	3D T1-weighted turbo field echo (TFE)	3T Philips Achieva	SagittalSagittal	220	0mm	1x1x1	NA	2.3ms	8.4ms	7	(12)

**FOR2107 Marburg**	3D T1-weighted magnetization prepared rapid acquisition gradient echo (MPRAGE)	3T Siemens Magnetom TiroTim syngo	Sagittal	176	0.5mm	1.0x1.0x1.0	900 ms	2.26 ms	1900 ms	9	(13)
**FOR2107 Muenster**	3D T1-weighted magnetization prepared rapid acquisition gradient echo (MPRAGE)	3T Siemens PRISMA	Sagittal	192	0mm	1.0x1.0x1.0	900 ms	2.28 ms	2130 ms	8	(13)
**Muenster**	3D fast gradient echo sequence	3T scanner Philips Gyroscan Intera	Coronal	320	0 mm	.5x.5x.5	814.5 ms	3.4 ms	7.4 ms	9	(14)
**NUIG**	3D T1-weighted magnetization prepared rapid acquisition gradient echo (MPRAGE)	1.5T Siemens Magnetom	Axial	256	0mm	0.45x0.45x0.9	600ms	4.38ms	1140 ms	15	(15; 16)
**Sao Paolo**	3D T1-weighted magnetization prepared rapid acquisition gradient echo (MPRAG	1.5T Siemens Espree	Sagittal	160	0mm	1.3 x 1.3 x 1.2	NA	3.65ms	2400 ms	8	(13)

	E)										
**Sydney**	3D T1-weighted turbo field echo (TFE)	3T Philips Achieva	Sagittal	180	1mm	1x1x1	NA	2.5ms	5.5ms	8	(17)
**TOP**	3D T1-weighted magnetization prepared rapid acquisition gradient echo (MPRAGE)	1.5T Siemens Sonata	Sagittal	160	0mm	1.33x0.94x1	1000 ms	3.93ms	2730 ms	7	(18; 19)
**UCSD**	3D T1-weighted fast spoiled gradient recall (FSPGR)	GE 3T Excite	Axial	176	1.2mm	1x1x1	600 ms	3.164 ms	2500 ms	8	
**UPenn**	3D T1-weighted magnetization prepared rapid acquisition gradient echo (MPRAGE)	3T Siemens Tim Trio	Axial	200	0mm	0.9x0.9x1	1100 ms	3.51ms	1810 ms	8	(20)

Supplementary Table S5: Fixed effects coefficients of logistic mixed-effects models assessing the effects of clinical covariates on probability of correct classification among BD participants. *Abbreviations:* AD – Antidepressants. AED - Antiepileptics; AIC - Akaike Information Criterion; FGA - First generation antipsychotics; Li – Lithium; SD – Standard deviation; SGA - Second generation antipsychotics

Predictors	*Dependent Variable - Correctly Classified*			
	Odds Ratio	*CI*	*Standard Error*	*p*
**Fixed Parts**				
(Intercept)	3.62	0.77 – 17.06	2.86	0.104
Sex	1.38	0.85 – 2.25	0.34	0.197
Age	1.4	1.05 – 1.88	0.21	**0.023**
Age o fOnset	1.03	0.73 – 1.47	0.19	0.849
Li	0.87	0.46 – 1.63	0.28	0.653
AED	1.73	1.07 – 2.78	0.42	**0.024**
FGA	0.95	0.29 – 3.04	0.56	0.927
SGA	0.67	0.37 – 1.23	0.21	0.197
AD	0.6	0.34 – 1.06	0.17	0.081
History of Psychosis	1.26	0.67 – 2.40	0.41	0.475
Full Diagnosis	0.58	0.27 – 1.22	0.22	0.148
MoodState	1.04	0.60 – 1.80	0.29	0.886
AIC	644.48			
-2 Log Likelihood	464.481			

Supplementary Table S6: Fixed effects coefficients of logistic mixed-effects models assessing the effects of clinical covariates on probability of being treated with anticonvulsants among BD participants. *Abbreviations:* AD – Antidepressants; AIC - Akaike Information Criterion; FGA - First generation antipsychotics; Li – Lithium; SD – Standard deviation; SGA - Second generation antipsychotics

Predictors	*Dependent Variable - Treatment with anticonvulsants*			
	Odds Ratio	*CI*	*Standard Error*	*p*
(Intercept)	1.44	0.31 – 6.71	1.13	0.643
Sex	0.95	0.49 – 1.83	0.32	0.879
Age	1.21	0.89 – 1.65	0.19	0.224
AgeofOnset	0.77	0.56 – 1.05	0.12	0.104
Li	0.39	0.19 – 0.80	0.14	**0.01**
FGA	0.83	0.19 – 3.70	0.63	0.808
SGA	1.21	0.72 – 2.06	0.33	0.468
AD	1.26	0.62 – 2.57	0.46	0.527
History of Psychosis	1.47	0.78 – 2.77	0.48	0.235
Full Diagnosis	0.96	0.51 – 1.84	0.32	0.914
MoodState	0.7	0.30 – 1.63	0.3	0.408
AIC	694.56			
-2 Log Likelihood	540.56			

Supplementary Figure SF1 Mixed-Effects Logistic Regression***,*** Predicted probabilities of correct classification based on covariates included in the original mixed-effects logistic regression model for subjects in the *Case* group. Each facet demonstrates the relationship between value of a predictor and the probability of correct classification (on the y-axis). Points within each facet show the distribution of subjects by predictor and correct classification. For example, those points clustered on the lower left are those for which the binary predictor was 0 and classification was *incorrect.* Those points on the upper right are those for which the binary predictor was 1 and the classification was *correct*. The slope of the black line represents the fixed effect coefficient for that variable.

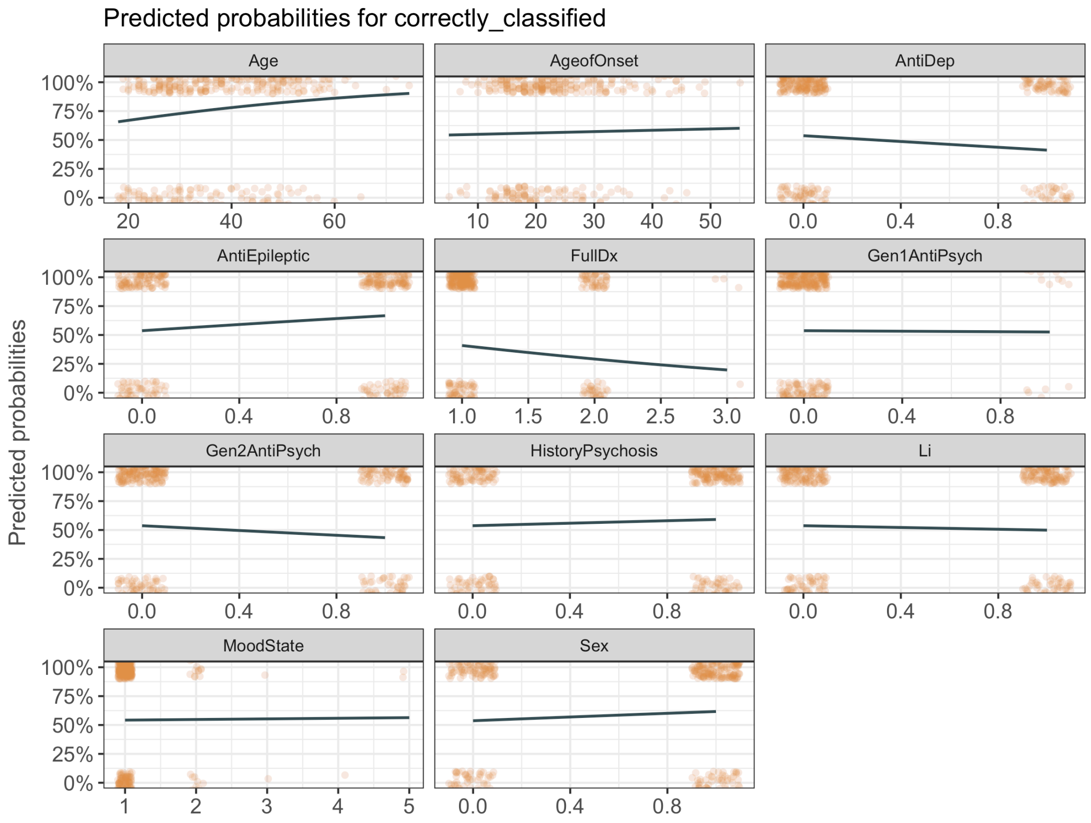

Supplementary References

(1) Rutter CM, Gatsonis C. A hierarchical regression approach to meta-analysis of diagnostic test accuracy evaluations. *Statistics in medicine* 2001; **20**(19):2865-2884.

(2) Bates D, MÃ¤chler M, Bolker B, Walker S. Fitting linear mixed-effects models using lme4. *Journal of Statistical Software* 2015; **67**(1):51.

(3) Satterthwaite FE. An Approximate Distribution of Estimates of Variance Components. *Biometrics Bulletin* 1946; **2**(6):110.

(4) Kuznetsova A, Brockhoff PB, Christensen RHB. lmerTest Package: Tests in Linear Mixed Effects Models. *Journal of Statistical Software* 2017; **82**(13).

(5) Pomarol-Clotet E, Canales-Rodriguez EJ, Salvador R, Sarro S, Gomar JJ, Vila F et al. Medial prefrontal cortex pathology in schizophrenia as revealed by convergent findings from multimodal imaging. *Mol Psychiatry* 2010; **15**(8):823-830.

(6) Amann BL, Canales-Rodriguez EJ, Madre M, Radua J, Monte G, Alonso-Lana S et al. Brain structural changes in schizoaffective disorder compared to schizophrenia and bipolar disorder. *Acta Psychiatr Scand* 2016; **133**(1):23-33.

(7) Caseras X, Lawrence NS, Murphy K, Wise RG, Phillips ML. Ventral striatum activity in response to reward: differences between bipolar I and II disorders. *Am J Psychiatry* 2013; **170**(5):533-541.

(8) van der Kouwe AJW, Benner T, Salat DH, Fischl B. Brain morphometry with multiecho MPRAGE. *Neuroimage* 2008; **40**(2):559-569.

(9) Hajek T, Calkin C, Blagdon R, Slaney C, Uher R, Alda M. Insulin resistance, diabetes mellitus, and brain structure in bipolar disorders. *Neuropsychopharmacology* 2014; **39**(12):2910-2918.

(10) Hajek T, Cullis J, Novak T, Kopecek M, Hoschl C, Blagdon R et al. Hippocampal volumes in bipolar disorders: opposing effects of illness burden and lithium treatment. *Bipolar Disorders* 2012; **14**(3):261-270.

(11) Hajek T, Bauer M, Simhandl C, Rybakowski J, O'donovan C, Pfennig A et al. Neuroprotective effect of lithium on hippocampal volumes in bipolar disorder independent of long-term treatment response. *Psychol Med* 2014; **44**(3):507-517.

(12) Elvsashagen T, Westlye LT, Boen E, Hol PK, Andersson S, Andreassen OA et al. Evidence for reduced dentate gyrus and fimbria volume in bipolar II disorder. *Bipolar Disord* 2013; **15**(2):167-176.

(13) Hibar DP, Westlye LT, Doan NT, Jahanshad N, Cheung JW, Ching CRK et al. Cortical abnormalities in bipolar disorder: an MRI analysis of 6503 individuals from the ENIGMA Bipolar Disorder Working Group. *Mol Psychiatry* 2017.

(14) Dannlowski U, Grabe HJ, Wittfeld K, Klaus J, Konrad C, Grotegerd D et al. Multimodal imaging of a tescalcin (TESC)-regulating polymorphism (rs7294919)-specific effects on hippocampal gray matter structure. *Mol Psychiatry* 2015; **20**(3):398-404.

(15) Emsell L, Langan C, Van Hecke W, Barker GJ, Leemans A, Sunaert S et al. White matter differences in euthymic bipolar I disorder: a combined magnetic resonance imaging and diffusion tensor imaging voxel-based study. *Bipolar Disord* 2013; **15**(4):365-376.

(16) Quigley SJ, Scanlon C, Kilmartin L, Emsell L, Langan C, Hallahan B et al. Volume and shape analysis of subcortical brain structures and ventricles in euthymic bipolar I disorder. *Psychiatry Res* 2015; **233**(3):324-330.

(17) Roberts G, Lenroot R, Frankland A, Yeung PK, Gale N, Wright A et al. Abnormalities in left inferior frontal gyral thickness and parahippocampal gyral volume in young people at high genetic risk for bipolar disorder. *Psychol Med* 2016; **46**(10):2083-2096.

(18) Rimol LM, Hartberg CB, Nesvag R, Fennema-Notestine C, Hagler DJ, Jr., Pung CJ et al. Cortical thickness and subcortical volumes in schizophrenia and bipolar disorder. *Biol Psychiatry* 2010; **68**(1):41-50.

(19) Haukvik UK, Westlye LT, Morch-Johnsen L, Jorgensen KN, Lange EH, Dale AM et al. In vivo hippocampal subfield volumes in schizophrenia and bipolar disorder. *Biol Psychiatry* 2015; **77**(6):581-588.

(20) Satterthwaite TD, Elliott MA, Ruparel K, Loughead J, Prabhakaran K, Calkins ME et al. Neuroimaging of the Philadelphia neurodevelopmental cohort. *Neuroimage* 2014; **86**:544-553.
